# Causal effect of the age at first birth with depression: a mendelian randomization study

**DOI:** 10.1186/s12920-024-01966-9

**Published:** 2024-07-24

**Authors:** Wanshu Guo, Yuanyuan Guo, Shaokang Song, Xuankai Huang, Yu Zhang, Aizhen Zhang, Fangrong Meng, Minghang Chang, Zhipeng Wang

**Affiliations:** 1 College of Animal Science and Technology, Northeast Agricultural University, Harbin, 150000 China; 2 Center for Bioinformatics, Northeast Agricultural University, Harbin, 150000 China; 3 Da Bei Nong Food Group , Breeding Center , Harbin, 150000 Heilongjiang China; 4 Branch of Animal Husbandry and Veterinary, Heilongjiang Academy of Agricultural Sciences, No. 2, Heyi St, Longsha District, Qiqihaer, 161005 China; 5 Yebio Bioengineering Co., Ltd of Qingdao, Qingdao, 266000 China

**Keywords:** Casual effect, Age at first birth, Depression, Mendelian randomization, Major depressive disorder, Postpartum depression

## Abstract

**Background:**

This study aimed to explore the causal relationship between age at first birth (AFB) and depression.

**Methods:**

Using the univariable Mendelian randomization (UVMR) and multivariable Mendelian randomization (MVMR) methods to examine the potential correlation between age at first birth (AFB) and major depressive disorder and postpartum depression. A public database was used to obtain the genome-wide association studies (GWAS) summary data. We put inverse-variance-weighted (IVW) as the primary method in Mendelian randomization (MR) analysis and used sensitivity analysis to confirm the robustness of our result.

**Results:**

We found a significant causal association between AFB and major depressive disorder by using the IVW algorithm (odd ratio [OR] 0.826; 95% confidence interval [CI] 0.793 − 0.861; *P* = 4.51 × 10^− 20^). MR-Egger, weighted median, simple mode and weighted mode method concluded the same result (*P* < 0.05). During the sensitivity analysis, the heterogeneity test (Q-value = 55.061, df = 48, *P* = 2.81 × 10^− 01^, I^2^ = 12.82%) and the leave-one-out plot analysis confirmed the stability of the results. The outcomes of the pleiotropy test (MR-Egger intercept = 8.932 × 10^− 3^. SE = 6.909 × 10^− 3^. *P* = 2.02 × 10^− 01^) and MR_PRESSO global test (*P* = 2.03 × 10^− 01^) indicated there is no pleiotropy.

**Conclusion:**

There is solid evidence that a higher age at first birth is associated with a lower risk of major depressive disorder.

## Introduction

The primary signs of depression are low mood, loss of interest, poor eating habits, and difficulty sleeping. The World Health Organization estimates that globally, an estimated 5% of adults suffer from depression [1], and the number is increasing quickly. The suicide rate among patients with depression is 20 times higher than that of the general population [2]. Major depressive disorder (MDD) and postpartum depression have relatively large impacts on humans. According to the definition from the DSM-5 (The Diagnostic and Statistical Manual of Mental Disorders, DSM) [3], major depressive disorder (MDD) is characterized by depressive mood or decrease in interest or pleasure for at least two weeks, almost most of the day, accompanied by at least five typical symptoms, including changes in sleep, changes in appetite, fatigue, difficulty concentrating, suicidal thoughts and so on. MDD has more complicated causes than typical depression, with higher rates of mortality and disability and a higher chance of recurrence [4]. As defined in DSM-5, postpartum depression is characterized by a prolonged duration of mood disturbances (which lasts for more than 2 weeks) occurring within the postpartum period (typically within 6 months after childbirth), accompanied by feelings of distress, sadness, low mood, and other negative emotions. About 20% of moms have postpartum depression, which also impacts how well their children develop [5, 6].

Depression is a complex disease with many influence factors, including genetics and environmental such as lifestyle habits. Howard et al. [7] identified 102 independent genetics variants and 269 genes associated with depression based on genome-wide association analysis (GWAS). Giannelis et al. [8] studied the link between family status and lifelong depression, it turns out that living with a spouse or partner was strongly associated with reduced odds of depression. Kendler et al. [9] found that stressful life events have a substantial causal relationship with the onset of episodes of MDD. According to the report [10–12], unhealthy lifestyle habits can also increase the risk of depression.

According to reports [13–17], there is a causal association between age at first birth (AFB) and depression. Some studies deem that there is a negative causal association between AFB and depression (Aitken et al., 2016; Mirowsky et al., 2002; Wang et al., 2023). Aitken et al. [13] found that teenage mothers are more likely than older mothers to have mental illness. According to Mirowsky et al. [14], there exists a general negative correlation between the age at first birth and feelings and signs of depression. Wang et al. [15] believe that early age at menarche (AAM), AFB, and age at first sexual intercourse (AFS) are risk factors for MDD. Ou et al. [16] found that the risk of postpartum depression decreased with the increase of AFB. However, some studies suggested that there is a positive association between AFB and depression. Astbury et al. [17] found the risk of postpartum depression increases when moms have their first child after age 34. Carlson et al., [18] found that women with first birth at both young (age 20 and younger) and older ages (after age 30) were positively associated with psychological distress. As mentioned above, current epidemiological studies on the relationship between AFB and depression have not reached a consistent conclusion. To investigate the possible causal relationship between AFB and depression, univariable Mendelian randomization (UVMR) and multivariable Mendelian randomization (MVMR) were used. Sensitivity analysis was also used in this study to ensure the accuracy and robustness of our results, including heterogeneity tests, leave-one-out analysis, pleiotropy tests, and MR-PRESSO global test.

## Methods

### Data sources

We put age at first birth (AFB) as exposure variable and depression as outcome variable in our research. The GWAS summary statistics f of AFB came from the study of Mills et al. [19], and their data were uploaded to the EBI database [20]. The study included 542,901 participants from France, Canada, and Germany and 9,702,772 single-nucleotide polymorphisms (SNPs). The GWAS Summary statistics data of major depressive disorder (MDD) and postpartum depression came from Wray et al. (2018) [21] in the Psychiatric Genomics Consortium (PGC) [22] and FinnGen Consortium [23], respectively. Wray’s study included 173,005 participants from Iceland, the USA, Denmark, and the UK and 13,554,550 SNPs. For postpartum depression study, it included 17,441 participants from the Finnish population and 16,376,215 SNPs. The confounding variables for current tobacco smoking came from Elsworth et al. [24], which included 462,434 participants from the UK and 9,851,867 SNP. For alcohol drinker status, it was came from Neale Lab genome-wide association meta-analysis (GWAS round 2) (http://www.nealelab.is/uk-biobank), which included 360,726 participants from the UK and 13,586,591 SNPs. All GWAS summary statistics were downloaded from the IEU OpenGWAS database [25, 26].

In this study, we only used summary statistics data to identify causal effets, which do not involve any private information about the sample. The GWAS summary statistics of the age at first birth (AFB), major depressive disorder (MDD), and postpartum depression were extracted from the EBI database (uploaded by Mills et al. (2021)), Psychiatric Genomics Consortium (uploaded by Wray et al. (2018)), and the FinnGen Consortium, respectively. Our results are used for academic research, not for commercial activities, which meet the rules and regulations of these databases.

The SNPs used as instrumental variables (IVs) in the MR analysis, which should satisfy the following assumptions: (1) ought to be closely associated with exposure; (2) ought to be unrelated to any confounding factors; and (3) ought to be directly associated with depression. So in order to meet the requirements as much as possible, we extracted significant SNPs related to AFB, according to *P* < 10^− 8^. We used linkage disequilibrium (LD) to minimize the number of IVs. When we set the smaller value of the R2, the number of IVs will be fewer, resulting in less confounding and pleiotropy. In this study, we set the threshold of LD as “distance > 10000 kb, R2 < 0.001” to filter the SNPs. The GWAS summary statistics of AFB, MDD and postpartum depression were exacted from the Psychiatric Genomics Consortium (uploaded by Wray et al. (2018)) and the FinnGen Consortium, respectively.

### Mendelian randomization

To calculate the causal effect between AFB and depression, we used five univariable Mendelian randomization (UVMR) algorithms, including MR-Egger, inverse variance-weighted (IVW), weighted median, weighted mode, and simple model.

MR-Egger method can yield an adjusted result by horizontal pleiotropy via the regression slope and intercept and a reasonably robust estimate independent of the validity of IVs [27, 28]. The IVW method can produce a reasonably stable and accurate causal evaluation by combining Wald estimates independent of the validity of IV [29, 30]. The weighted median method is that the Mendelian randomization (MR) estimates generated using each instrument weighted for the inverse of their variance are arranged in order by the weighted median method. A single MR estimate with confidence intervals computed by parametric bootstrap is obtained by choosing the median result [31]. When the most extensive collection of instruments with consistent MR estimates is valid, the weighted mode based causal estimate consistently calculates the true causal impact [32]. We put the IVW method as the primary method used in this paper; the other four methods were used as complementary analyses. The simple Mode method uses the causal effect estimates for individual SNPs to form clusters, the causal effect estimate is then taken as the causal effect estimate from the largest cluster of SNPs [33]. In order to better understand the relationship between AFB and depression and to increase the reliability of the results, multivariable Mendelian randomization (MVMR) method was also used in this paper. MVMR is an extension of univariate MR. It can estimate the causal relationship between multiple exposures and a single outcome. It is particularly used in situations where multiple exposures are related.

The effect of lifestyle habits on depression may be a potential confounding factor. We selected alcohol and smoking as the potential confounding factors to perform MVMR analysis with AFB on the risk of depression.

Univariable Mendelian randomization (UVMR) and multivariable Mendelian randomization (MVMR) analyses were performed in R (version 4.2.3) using the “TwoSampleMR” version 0.5.6 [34]. The exposure variable and the outcome have a significant causal association (*P* < 0.05).

### Sensitivity analysis

Four methods were used for sensitivity analysis: including heterogeneity test, pleiotropy test, MR-PRESSO test, and leave-one-out analysis. Heterogeneity test was measured by two methods, including Cochran’s Q test and I^2^ statistics (I^2^ = 100% × (Q − df)/Q, where Q is the Cochran’s Q test value, and df is the number of instrumental variables minus one) [35]. if the P value (Q-statistic) < 0.05 or I2 > 50%, which suggests there should exit heterogeneity [36]. The MR pleiotropy residual sum and outlier test (MR_PRESSO) [37] and the intercept from the MR-Egger method ^[14]^ were used to check for directional pleiotropy; *P* < 0.05 indicates exits pleiotropy. Leave-one-out analysis was tested by removing the SNPs one by one [38], and it was judged by observing the plot.

## Results

### UVMR analysis of age at first birth with depression

A total of 50 SNPs, and 55 SNPs were chosen as instrument variables (IVs) for major depressive disorder (MDD), and postpartum depression to do univariable Mendelian randomization (UVMR) analysis, respectively.

**Fig. 1:**
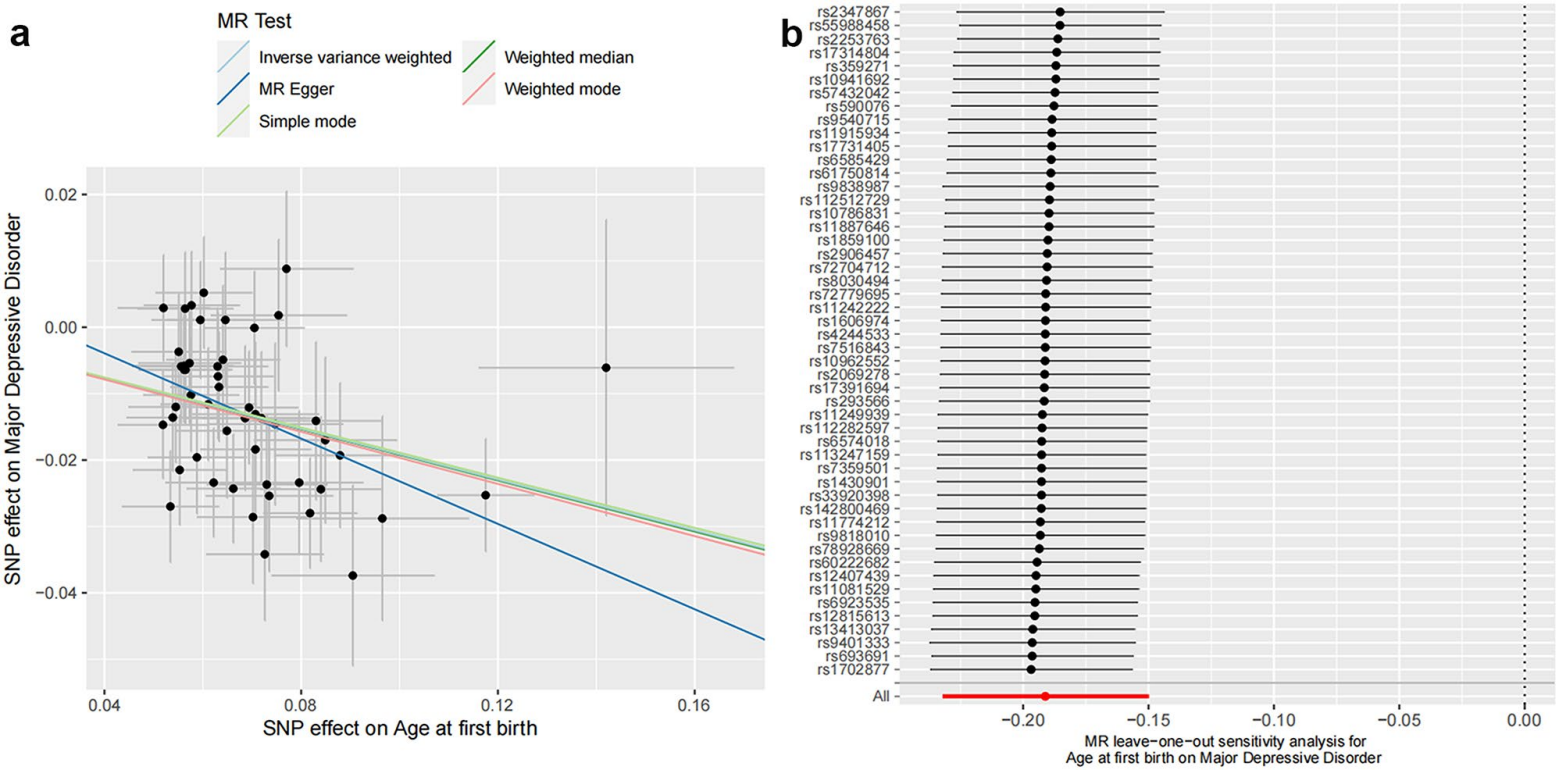
**a** Scatter plot for AFB on the risk of MDD. **b** Leave-one-out plot for AFB on MDD

The IVW algorithm showed that there exists a significant negative causal association between AFB and MDD (OR = 0.826; 95% CI, 0.793 − 0.861; *P* = 4.51 × 10^− 20^). Table 1 shows results of four other algorithms are also significant (*P* < 0.05). Through the scatter plot (Fig. 1a), we can draw the same conclusion as the IVW algorithm. Every sensitivity analysis method revealed no impact on our outcome. Leave-one-out plot (Fig. 1b) and heterogeneity test (Q-value = 55.061, df = 48, *P* = 2.81 × 10^− 01^, I^2^ = 12.82%), pleiotropy test (MR-Egger intercept = 8.932 × 10^− 3^, SE = 6.909 × 10^− 3^, *P* = 2.02 × 10^− 01^) and MR_PRESSO global test (*P* = 2.03 × 10^− 01^) also came to the same conclusion. Every sensitivity analysis result confirmed the robustness of the results.

**Fig. 2:**
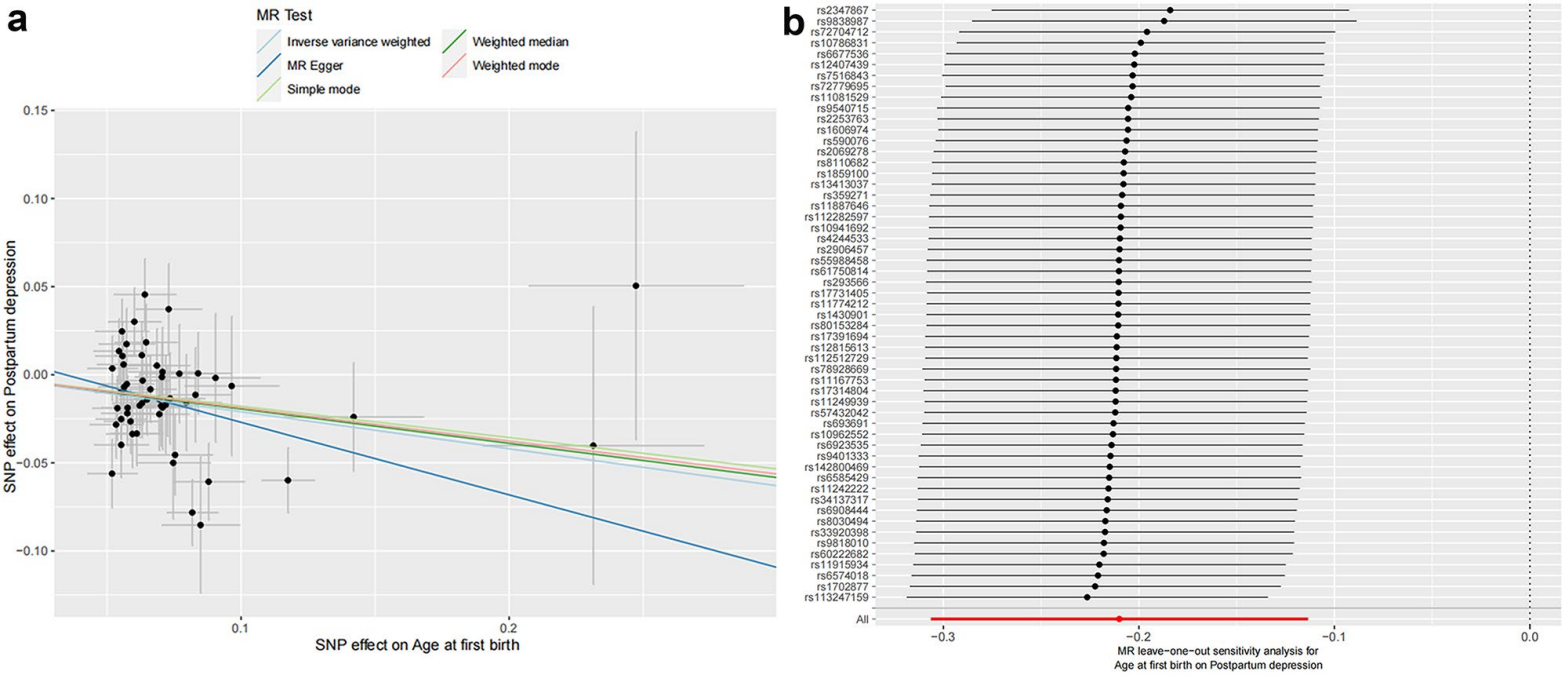
**a** Scatter plot for AFB on the risk of postpartum depression. **b** Leave-one-out plot for AFB on postpartum depression (B)

The IVW approach indicated that AFB and postpartum depression have a significant negative causal association (OR = 0.810; 95% CI, 0.736 − 0.892; *P* = 1.92 × 10^− 05^). Table 1 showed that the results of MR-Egger and weighted median are significant (*P* < 0.05), but weighted mode and simple mode are not (*P* > 0.05). The causal association between AFB and postpartum depression can also observed by scatter plot (Fig. 2a). During the sensitivity analysis, no significant SNPs were found in the leave-one-out plot (Fig. 2b). Heterogeneity test (Q-value = 74.043, df = 54, *P* = 3.60 × 10^− 02^, I^2^ = 27.07%) indicated that there exists heterogeneity. The results of the pleiotropy test (MR–Egger intercept = 1.420 × 110^− 2^, SE = 1.371 × 10^− 2^, *P* = 3.05 × 10^− 01^) and MR_PRESSO global test (*P* = 3.90 × 10^− 02^) showed contrast, suggesting the possibility of horizontal pleiotropy. Based on the results of sensitivity analysis, we think that there is no causal association between AFB and postpartum depression since heterogeneity and pleiotropy would go against the MR approach’s assumptions.

A reverse Mendelian randomization (MR) analysis was also performed. We put depression as exposure variable and age at first birth (AFB) as outcome variable. For major depressive disorder (MDD), 4 instrument variables (IVs) were extracted. Inverse variance weighted (IVW) algorithm indicated that there is no significant association between MDD and AFB (OR = 0.794; 95% CI = 0.477 − 1.323; *P* = 3.76 × 10^− 01^). According to Table 2, the results of MR-Egger, weighted median, simple mode and weighted mode algorithm was still insignificant (*P* > 0.05). Moreover, the outcomes of sensitivity analysis further indicated that there were no heterogeneity (Q-value = 8.24, df = 2, *P* = 6.62 × 10^− 02^, I^2^ = 24.27%) and pleiotropy (MR–Egger intercept = 1.00 × 10^− 2^, SE = 2.64 × 10^− 2^, *P* = 3.05 × 10^− 01^), which proves the stability and reliability of the results. For postpartum depression, 3 instrument variables (IVs) were extracted. IVW algorithm showed that there is a significant negatively causal association between postpartum depression and AFB (OR = 0.285; 95% CI, 0.158 − 0.514; *P* = 7.69 × 10^− 01^). Table 2 shows the results of weighted median algorithm are also significant (*P* < 0.05), but the results of MR Egger, simple mode and weighted mode are not (*P* > 0.05). However, the results of the sensitivity analysis showed heterogeneity (Q-value = 25.66, df = 2, *P* = 2.68 × 10^− 06^, I^2^ = 7.79%). Accordingly, we believed that there was no causal association between them when we put postpartum depression as an exposure variable.

**Table 1:** Two MR results of age at first birth on the risk of depression

Variable outcome	nSNP	OR	95%CI	*P*
Major depressive disorder				
MR Egger	50	0.725	0.593–0.887	3.00 × 10^− 03^
Weighted median	50	0.825	0.777–0.877	4.59 × 10^− 10^
IVW	50	0.826	0.793–0.861	4.51 × 10^− 20^
Simple mode	50	0.828	0.720–0.952	1.06 × 10^− 02^
Weighted mode	50	0.821	0.726–0.930	3.17 × 10^− 03^
**Postpartum depression**				
MR Egger	55	0.662	0.446–0.982	4.54 × 10^− 02^
Weighted median	55	0.823	0.728–0.930	1.90 × 10^− 03^
IVW	55	0.810	0.736–0.892	1.92 × 10^− 05^
Simple mode	55	0.837	0.611–1.145	2.71 × 10^− 01^
Weighted mode	55	0.829	0.618–1.110	2.14 × 10^− 01^

*Notes* OR, odds ratio; nSNP, number of single-nucleotide polymorphism; CI, confidence interval

**Table 2:** Reverse MR results of depression on age at first birth

Variable exposure	nSNP	OR	95%CI	*P*
**Major depressive disorder**				
MR Egger	4	1.670	0.081–34.339	7.71 × 10^− 01^
Weighted median	4	1.012	0.791–1.295	9.22 × 10^− 01^
IVW	4	0.794	0.477–1.323	3.76 × 10^− 01^
Simple mode	4	1.058	0.780–1.401	7.00 × 10^− 01^
Weighted mode	4	1.058	0.829–1.351	6.90 × 10^− 01^
**Postpartum depression**				
MR Egger	3	0.670	0.003–140.730	9.07 × 10^− 01^
Weighted median	3	0.362	0.195–0.674	1.34 × 10^− 03^
IVW	3	0.285	0.158–0.514	2.93 × 10^− 05^
Simple mode	3	0.374	0.219–0.639	6.91 × 10^− 02^
Weighted mode	3	0.366	0.227–0.590	5.42 × 10^− 02^

*Notes* OR odds ratio; nSNP, number of single-nucleotide polymorphism; CI, confidence interval

### MVMR analysis of age at first birth with depression

Smoking and alcohol are presented as potential confounding factors for MVMR analysis in this paper. Each of them is added as a separate factor to the MVMR analysis. AFB is the protective factor for MDD when smoking is controlled, according to the MVMR results (OR = 0.864; 95% CI, 0.799 − 0.929; *P* = 8.29 × 10^− 06^). There was compelling evidence of a direct effect of AFB on the incidence of MDD after controlling for alcohol (OR = 0.843; 95% CI, 0.782 − 0.904; *P* = 2.87 × 10^− 08^).

## Discussion

Mendelian randomization (MR) analysis, as a method for causal association inference, has been widely applied across various epidemiological studies [39–41]. In this study, we performed a bidirectional MR analysis to identify the causal association between age at first birth (AFB) and major depressive disorder (MDD) and postpartum depression, respectively. According to the results based on five algorithms, there was a significant causal association between AFB and MDD, and the sensitivity analysis further confirmed the robustness and dependability of the study findings.

Compared to conventional research methodologies, Mendelian randomization (MR) offers numerous advantages for determining causal relationships. First, elements deemed to be uncontrollable may impact the trial findings when using traditional experimental or research procedures. To investigate the connection between exposure and outcome variables, MR techniques use genetic variation data as a link [42]. Genetic variations can be employed as potential instrument variable (IV) since they are present at birth, do not alter during life, and are unaffected by various confounding factors, including acquired environments. Second, With the development of sequencing technology, a large number of variant sites associated with human diseases have been detected, which provides a great convenience for the utilization of MR methods, and MR methods are not only easy to use, but also can help to discover potential causal associations between diseases. Third, whereas human genetic information can change exposure variables, exposure variables do not modify human DNA sequences. So the MR approach is unaffected by causal inversion.

There have been numerous studies on the relationship between AFB and MDD. Wang et al. [15] found that lower AFB is one of the key risk factors of MDD. Cai et al. [43] suggested that an increase in AFB would reduce the risk of MDD. He et al. [44] believed that earlier AFB could significantly predict a higher risk of MDD. Ohi et al. [45] found that people with MDD commonly had a younger AFB. Ni et al. [46] believed that the younger the AFB, the higher the risk of MDD. This study also found a negative causal correlation between AFB and MDD through the analysis method of MR. However, according to the study of McMahon et al. [47], maternal age was not related to the prevalence of major depression episodes. This is inconsistent with the conclusions reached in this study, which may be caused by the difference in population, sample size, analysis methods, and so on.

Studies on the relationship between AFB and postpartum depression have shown positive or negative correlations. Ou et al. [16] found that the risk of postpartum depression decreased with the increase of AFB. Zuo et al. [48] found that increased AFB will lead to a decreased probability of postpartum depression. But Astbury et al. [13] found that the risk of postpartum depression increases beyond the age of 34 at first birth. Our results shown it there may be a negative causal correlation between AFB and postpartum depression through MR analysis.

The etiology of MDD is highly intricate, and it is not only regulated by genes and genetics, but also related to other factors such as stress, human behavior, and so on. Huo et al. [49]and Wray et al. [21] employed meta-analysis and genome-wide association analysis (GWAS) identified a large number of candidate genes related to MDD, including Solute carrier family 25 member 37 (*SCL25A37*), Raftlin family member 2 (*RFTN2*), Transcription Factor 4 (*TCF4*), and Butyrophilin subfamily 1 member A1 (*BTN1A1*) genes. Kendler et al. [9] believed that there was a sustained causal correlation between life stress and the onset of MDD. Covey et al. [50] and Rosenthal et al. [51] found that smoking and drinking alcohol can increase the risk of MDD. Ni et al. [46] and Zhao et al. [52] suggested that younger AFS can increase the risk of MDD. In summary, there are numerous contributing factors to MDD, which warrants additional research in the future for clarification.

This study has some strengths that the other do not. We use a variety of sensitivity analysis methods to verify that our results are robust and reliable, and our research will only be conclusive if all sensitivity analysis methods are satisfied. Therefore, the conclusion of this study can provide strong evidence for the association between AFB and depression. In addition, compared with other research methods, the method of bidirectional Mendelian randomization in this study can effectively avoid the problems of reverse causality and interference factors, and improve the accuracy of causal inference.

This study has some limitations. First, the criteria for instrumental variables (IVs) were relatively strict, hence many variation sites related to AFB were excluded, which may have led to the effect of some variation sites not reflecting in the results of this study. Second, in the two-sample MR analysis, although there was a strong correlation between IVs and exposure variables, if the IVs had a small effect size, the association between exposure and outcome variables could be underestimated. Third, our discovery sample mainly consisted of the European population, and diversifying the study population would help verify its applicability to other ethnic groups.

## Conclusions

In conclusion, our results demonstrated that there exists a negative causal association between AFB and major depressive disorder. When the age at first birth increased in humans, the risk of major depressive disorder decreased significantly. Nevertheless, the reason for the causal association between AFB and major depressive disorder is unknown, there may be some molecular regulatory mechanism or environmental factors between them. More detailed studies of the causal association between AFB and major depressive disorder are needed in the future. We hope this paper will add some fresh perspectives and reference values to the field of depression research.

## Data Availability

Full summary statistics on AFB (id: ebi-a-GCST90000050), major depressive disorder (id: ieu-a-1188), smoking (id: ukb-b-223) and alcohol consumption (id: ukb-d-20117_2) were obtained from GWAS summary datasets generated by many different consortia and were deposited in the MR-Base database (https://gwas.mrcieu.ac.uk/). Full summary statistics for postpartum depression (id: finn-b-O15_POATPART_DEPR) are available at FinnGen Biobank (https://r9.finngen.fi/).
